# Evaluation of prophylaxis protocols and spatial distribution of humans wounded by bats: a descriptive study, Palmas, 2013-2023

**DOI:** 10.1590/S2237-96222026v35e20250557.en

**Published:** 2026-03-16

**Authors:** Thais Cristine Rodrigues Freitas, Anderson Brito Soares, Benta Natânia Silva Figueiredo

**Affiliations:** 1 Fundação Escola de Saúde Pública de Palmas, Palmas, TO, Brazil; 2 Secretaria Municipal de Saúde de Palmas, Unidade de Vigilância e Controle de Zoonoses, Palmas, TO, Brazil

**Keywords:** Rabies Care, Lyssavirus, One Health, Health Surveillance, Geographic Mapping, Atención Antirrábica, Lyssavirus, Salud Única, Vigilancia Sanitaria, Mapeo Geográfico

## Abstract

**Objective:**

To evaluate prophylaxis prescriptions in cases of bat-related accidents involving humans and the spatial distribution of accidents and bats captured by the zoonosis surveillance unit of Palmas, Tocantins, from 2013 to 2023.

**Methods:**

This was a retrospective descriptive study of prophylaxis prescriptions during initial care of bat-related accidents involving humans, held on electronic medical records (e-SUS) and on the case reporting form (via the Notifiable Health Conditions Information System) for rabies treatment. The following variables were analyzed: patient sex and age, type of exposure, site and characterization of the wound, history of rabies treatment, treatment prescribed according to the current protocol, indication of rabies antiserum and interruption of treatment. The location of patients treated and bats captured for laboratory diagnosis was georeferenced to produce distribution maps.

**Results:**

Of 136 reported cases, 68.4% (93/136) of initial care adequately followed the current prophylaxis protocol. The Southeast region of the municipality presented the highest concentration of reported residents (37/123). Between 2015 and 2023, 133 bats were captured from residences and analyzed for the presence of the rabies virus, of which 2.3% (3/133) were reactive, found in the Northwest, Southwest and Southern regions of the municipality.

**Conclusion:**

The study highlighted the relevance of epidemiological surveillance in monitoring prophylaxis protocols and care for accidents involving bats, given the inconsistencies in initial care. The importance of continuous training for health professionals stood out. Detection of the virus in urban bats evidenced its circulation and reinforced the need for integrated interinstitutional actions.

## Resumen

**Objetivo:**

Evaluar las prescripciones profilácticas en casos de accidentes con murciélagos que involucran a humanos y la distribución espacial de los accidentes y los murciélagos capturados por la unidad de vigilancia de zoonosis de Palmas, Tocantins, entre 2013 y 2023.

**Métodos:**

Estudio descriptivo retrospectivo de las prescripciones profilácticas durante la atención inicial de accidentes con murciélagos que involucran a humanos, registradas en historias clínicas electrónicas (e-SUS) y en el formulario de notificación (a través del Sistema de Información de Enfermedades de Notificación Obligatoria) para el tratamiento de la rabia. Se analizaron las siguientes variables: sexo y edad del paciente, tipo de exposición, localización y características de la herida, antecedentes de tratamiento antirrábico, tratamiento prescrito según el protocolo vigente, indicación de antisuero antirrábico e interrupción del tratamiento. La ubicación de los pacientes tratados y de los murciélagos capturados para diagnóstico de laboratorio se georreferenció para generar mapas de distribución.

**Resultados:**

De los 136 casos notificados, el 68,4% (93/136) de la atención inicial siguió adecuadamente el protocolo de profilaxis vigente. La región Sudeste del municipio presentó la mayor concentración de residentes notificados (37/123). Entre 2015 y 2023, se capturaron 133 murciélagos en domicilios y se analizaron para detectar la presencia del virus de la rabia; de estos, el 2,3 % (3/133) resultaron reactivos, encontrados en las regiones Noroeste, Suroeste y Sur.

**Conclusión:**

El estudio destacó la relevancia de la vigilancia epidemiológica para el seguimiento de los protocolos de profilaxis y la atención de accidentes con murciélagos, dadas las inconsistencias en la atención inicial. Se enfatizó la importancia de la capacitación continua para los profesionales de la salud. La detección del virus en murciélagos urbanos evidenció su circulación y reforzó la necesidad de acciones interinstitucionales integradas.

**Ethical aspects**

This research respected ethical principles, having obtained the following approval data:

Research ethics committee: Fundação Escola de Saúde Pública de Palmas

Opinion number: 6,901,095

Approval date: 21/6/2024

Certificate of submission for ethical appraisal: 77352424.4.0000.9187

Informed consent form: Not applicable.

## Introduction 

Rabies is a fatal zoonotic infectious disease that affects the central nervous system of mammals and its etiological agent is a virus of the Rhabdoviridae family and the Lyssavirus genus (1). It is a disease of extreme concern for public health, due to its unfavorable prognosis and the wide genetic diversity of its etiological agent (2).

The virus enters the body through inoculation via saliva from an infected mammal, mainly through bitten, scratched or licked mucous membranes (3). The rabies transmission chain can be divided into four cycles: urban, rural, terrestrial wild and aerial wild, the latter referring to all species of bats, whether hematophagous (vampire) or not (4).

In Latin America, following efforts undertaken by the Pan American Health Organization (PAHO) since 1983, the incidence of dog-borne rabies has been reduced by approximately 98%, with bats being, in 2024, the main reservoirs involved in its transmission (5). In Brazil, following the same pattern, cases of human rabies transmitted by animals in the wild cycle are highlighted, given that the last human case caused by the antigenic variant of rabies virus transmitted by dogs (AgV-1) was recorded in 2015 (6,7).

Natural factors, such as animal habitats, reservoirs and virus circulation, combined with the direct impacts of human activities on the environment, play a crucial role in the epidemiological study of rabies (8). The state of Tocantins has great biodiversity, marked by the vegetation of the Amazon rainforest and the Cerrado. Habitat fragmentation and loss actions resulting from agriculture, such as urban expansion, mining and highway construction, can influence the transmission of rabies in the state, since Tocantins has 48% of the bat species identified in Brazil (9,10).

Between January 2010 and January 2025, 51 cases of human rabies were reported in Brazil, with 22 (43.1%) of these caused by bats. During this period, two deaths were recorded in Tocantins, in the municipalities of Ponte Alta do Tocantins and Alvorada, in 2017 and 2024, respectively, both caused by antigenic variant 3 (AgV-3), transmitted by hematophagous bats (11).

Due to the case fatality ratio of approximately 100% of cases in humans and the absence of specific treatment, the importance of the correct performance and execution of rabies virus pre-, post- and re-exposure prophylaxis protocols is evident (12). Prophylaxis is based on the potential risk of infection by the virus and should be adopted through case analysis, complete patient history and recording of the rabies data (13).

The prophylaxis protocol currently recommended by the Ministry of Health consists of administering rabies vaccine with or without the use of rabies antiserum or human rabies immunoglobulin, depending on the situation observed. All cases of accidents involving bats and other wild animals should be considered serious accidents, with follow-up and monitoring of these patients, so that cases of treatment dropout and insufficient prophylaxis administration are avoided (14).

The objective of this study was to evaluate prophylaxis prescriptions in cases of bat-related accidents involving humans, and the spatial distribution of accidents and bats captured by the zoonosis surveillance unit in Palmas, Tocantins, from 2013 to 2023.

## Methods 

### Design 

This was a retrospective descriptive study of the prophylactic measures prescribed for cases for whom bat-related accidents were reported. Georeferencing of their respective residences and location of bat captures in Palmas, was used, covering the period from January 2013 to December 2023.

### Setting 

Palmas is the capital of the state of Tocantins and is part of the Legal Amazon (Amazônia Legal) region. Is located 260 meters above sea level in south-central region of the state. It is bordered to the west by the lake formed by the Luís Eduardo Magalhães hydroelectric dam and to the east by the Carmo and Lajeado mountain ranges. In 2022, with 302,692 inhabitants, it had a territorial area of ​​2,227.329 km^2^ and a population density of 135.90 inhabitants/km^2^. Temperatures are high throughout the year with low variation and a well-defined seasonal distribution, with a rainy season from October to April, marked by light to moderate winds, and a dry season from May to September.

**Figure 1:**
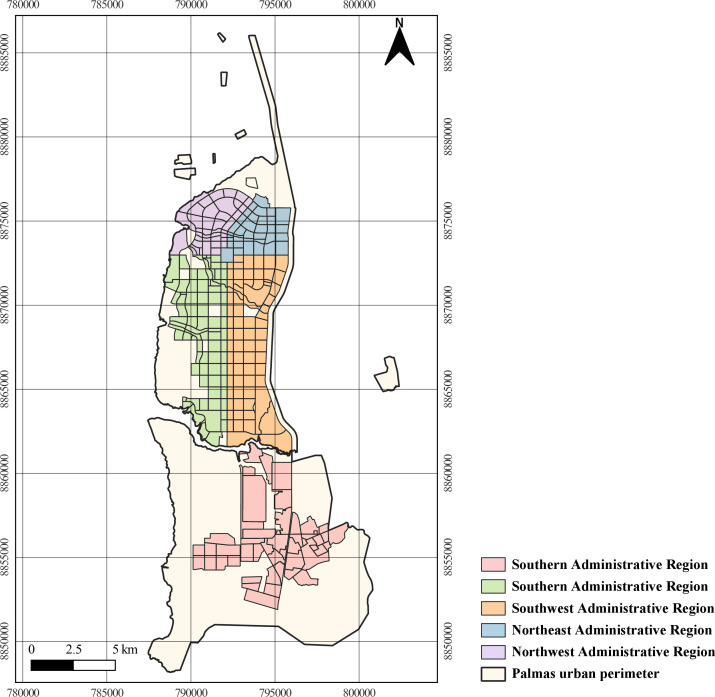
Administrative regions of the urban perimeter. Palmas, 2024

The city is characterized by vegetation areas within the urban perimeter, forming an ecological corridor, with the presence of parks and reserve areas. Administratively, the municipality has five regions: South, Southwest, Southeast, Northwest and Northeast (15,16) (Figure 1).

### Data source

Data were collected by exporting human anti-rabies treatment records reported at the municipal level, specifically for bat-related accidents, held on the National Notifiable Health Conditions Information System. To supplement relevant patient care information, we also used data from the Citizen Electronic Health Record e-SUS panel and the City Health Department.

### Data variables and analysis 

Data on administration of prophylactic care were exported using TabWin software (version 4.13) and subsequently organized into spreadsheets before undergoing descriptive analysis. Medical records with incorrect information and missing complete addresses, which prevented georeferencing, were excluded.

The following variables were analyzed in order to analyse the prescribed prophylactic measures: patient sex and age, type of exposure, site and characterization of the wound, history of antirabies treatment, treatment prescribed according to the current protocol, indication for antirabies serum and interruption of treatment (13).

For the purposes of georeferencing, we used information regarding the neighborhood, street, house number, address details, and zone of the reported cases, as well as the latitude and longitude of the places where bats were captured. When addresses were incomplete, we used the midpoint of the thoroughfare in question for georeferencing. 

The information about the locations was organized into spreadsheets and exported to QGis software (version 3.28.11-Firenze). This enabled the creation of heat maps using the kernel density estimator, a tool that calculated the density of points in geographic space, where, through color gradations, areas with a higher concentration of specific events were highlighted (17).

Samples with positive rabies virus test results were georeferenced and underwent descriptive analysis.

## Results 

**Figure 2:**
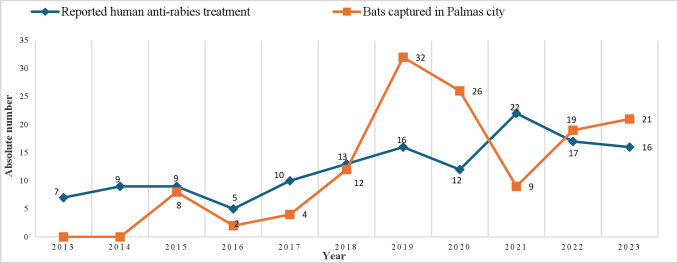
Absolute numbers of reported cases of human anti-rabies treatment due to bat wounds from 2013 to 2023 (n=136) and of bats captured from 2015 to 2023 (n=133). Palmas, 2024

In the period analyzed, 136 reported cases of human anti-rabies treatment related to bat wounds were recorded in Palmas (annual average = 12.36 cases). We found that 2021 was the year that concentrated the highest number of reported cases, corresponding to 16.2% (22/136) of the total (Figure 2). 94.9% (129/136) of reported cases originated from urban areas. Male patients were the most affected, accounting for 58.8% (80/136) of cases, with the 20-34 age group accounting for 35.3% (48/136), followed by the 35-49 age group with 29.4% (40/136), 50-64 years with 18.4% (25/136), 15-19 years with 5.1% (7/136), 65-79 years with 3.7% (5/136), 1-4 years with 2.9% (4/136), 5-9 years with 2.2% (3/136), 10-14 years with 2.2% (3/136), and 80+ years with 0.7% (1/136) of reported cases.

The most common type of accident with risk of exposure to the rabies virus was bat bites (81.6%), occurring mainly in the hands and feet (66.9%). The wounds were mostly described as single in 74.3% and deep in 53.0% of cases (Table 1).

**Table 1:** Characteristics of accidents related to reported cases of human anti-rabies treatment due to bat wounds from 2013 to 2023. Palmas, 2024 (n=136)

Characteristics	n (%)
**Type of exposure to the rabies virus**	
Indirect contact	2 (1.5)
Scratch	17 (12.5)
Licking	3 (2.2)
Bite	111 (81.6)
Other	4 (2.9)
Site	
Mucous membrane	2 (1.5)
Head/neck	12 (8.8)
Hands/feet	91 (66.9)
Torso	9 (6.6)
Upper limbs	9 (6.6)
Lower limbs	12 (8.8)
Wound	
Single	101 (74.3)
Multiple	25 (18.4)
No wound	10 (7.4)
**Type of wound**	
Deep	72 (53.0)
Surface	53 (39.0)

Analysis of initial recorded care showed that 68.4% (93/136) of cases adequately followed the current prophylaxis protocol. The remaining cases (43/136) presented unsatisfactory prescriptions at the first consultation: absence of rabies antiserum prescription in 44.2% (19/43), adoption of an obsolete protocol in 27.9% (12/43), prescription of insufficient doses of rabies vaccine in 9.3% (4/43), inadequate re-exposure protocol in 7.0% (3/43), absence of prescription information in 7.0% (3/43), observation of the animal in 2.3% (1/43), and decision not to treat in 2.3% (1/43).

One hundred percent of prescriptions classified as unsatisfactory underwent intervention by the municipal epidemiological surveillance service, via the Palmas City Health Department Human Anti-Rabies Care Technical Area, to adapt them to the current protocol.

Of the total number of cases reported, 10.3% (14/136) dropped out of the prescribed treatment. In turn, 1.5% (2/136) of the cases were closed as treatment dropout on the case reporting form, even though they completed the regimen. Active searching for patients classified as treatment dropout was carried out in 100.0% of cases.

**Figure 3:**
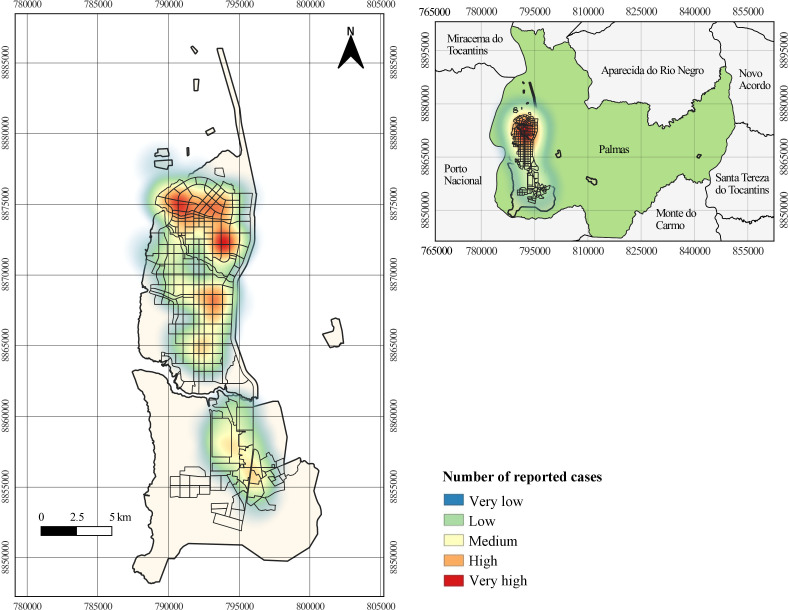
Reported cases of patients attacked by bats from 2013 to 2023, with the highest number in the areas marked in red. Palmas, 2024 (n=123)

A total of 123 addresses were located and interpreted according to the kernel density estimator (Figure 3). The Southeast administrative region showed the highest concentration of reported cases (37/123), followed by the South (29/123), Northwest (24/123), Southwest (19/123) and Northeast (14/123) regions.

Between 2015 and 2023, 133 bats were captured from residences in the municipality and analyzed for the presence of the rabies virus, of which 2.3% (3/133) tested positive, with 1 in the Northwest in 2018, 1 in the Southwest and 1 in the South in 2019.

**Figure 4:**
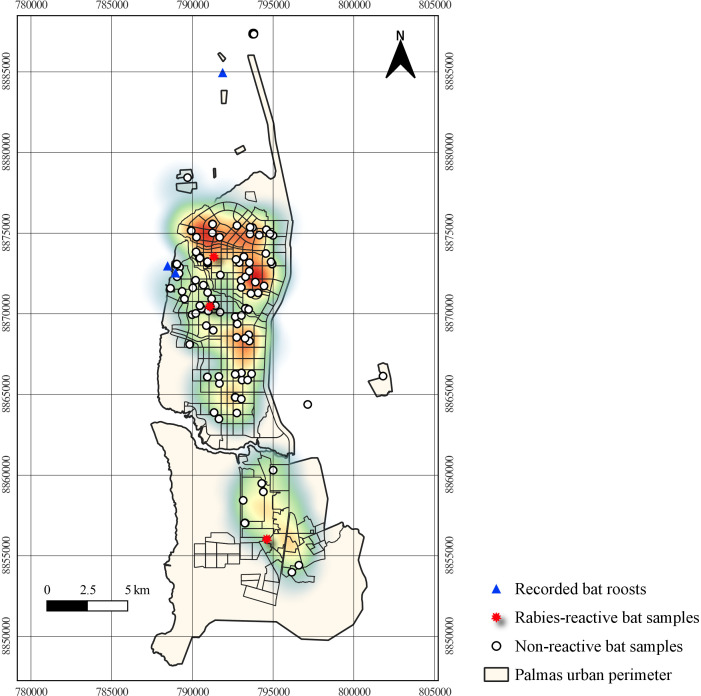
Distribution of points where bats were captured for laboratory diagnosis and their recorded roosts, superimposed on the heat map of addresses of patients attacked by bats in the period 2013-2023. Palmas, 2024 (n=133)

Thirty-five of the bats captured were from the Southeast administrative region; 31 from the Southwest; 24 from the Northwest; 21 from the Northeast; 14 from the South; and 8 from the rural area. Regarding bat roosts recorded within the urban perimeter of Palmas, 2 were georeferenced, both found in the Southwest region (Figure 4).

## Discussion 

The analysis of the records of human anti-rabies treatment due to bat wounds showed a predominance of male patients, those aged 20 years and older, and urban area residents. This sociodemographic pattern corroborated the literature, which described a similar profile in treatments performed in Brazilian state capitals (18).

When evaluating the prophylaxis prescription protocols followed during first care, we found that the protocols were adequate in 68% of cases. However, part of them presented discrepancies, since such cases should have been handled as serious accidents, with serum vaccination being paramount in post-exposure situations (13).

Although most prescriptions adequately followed recommendations in accident situations involving risk of rabies virus transmission, errors were reported in initial care when compared to the current protocol, such as the exclusive use of vaccine or even decision not to treat (19,20). Cases of prophylaxis regimen interruption and/or dropout were described, as well as records duplicated in different health centers, which may indicate weaknesses during the filling out of case reporting forms and compliance with the current protocol (20). These data were important for the planning and execution of the service offered, since, between 2002 and 2012, in 80% of human rabies deaths caused by wild mammals in Brazil, there was no type of anti-rabies prophylaxis or it was administered inadequately (21).

Evidence has shown that the high turnover of doctors and nurses, coupled with insufficient ongoing educational activities, has contributed to insecurity in treatment decision-making and prescribing by health professionals (22,23).

This study found that epidemiological surveillance acted effectively, adapting all protocols in a timely manner and actively searching for all treatment dropout or treatment interruption cases. These findings reinforced the relevance of surveillance, while also highlighting the need to strengthen the continuous training of health professionals and integration between animal and human surveillance. This allowed for the incorporation of intersectoral strategies, aligned with the “One Health” perspective, in order to ensure greater coherence and efficiency in response actions.

Due to changes in the epidemiological profile of human rabies, the need has arisen for improved surveillance strategies to prevent this disease, especially in areas with higher risk of exposure to bats (6). Of the 182 bat species recorded in Brazil, 46% were found in urban environments. Favorable factors in this environment for the continuing presence of bat colonies, such as ease of finding artificial roosting places and availability of food, demonstrated the predisposition for the formation of colonies in these locations (24). In turn, the predominance of attacked patients residing in the urban area of ​​Palmas also highlighed the process of territorial expansion of the state capital. In turn, the predominance of attacked patients residing in the urban area of Palmas also highlighed the process of territorial expansion of the state capital, favoring overlapping between anthropic environments and bat shelter and feeding areas (25)

Insectivorous bat species are attracted to city lighting, as insects gather around it, in addition to frugivorous, phytophagous and nectarivorous bat species, which are attracted to trees (24). The presence of a large colony of insectivorous bats of the Molossidae family was reported in the urban area of ​​Palmas, in a roost located on the Governor José Wilson Siqueira Campos bridge – the same roost was georeferenced in this study (Figure 4). This information highlighted the need to consider the existence of these animals in the urban environment, their population sustainability, the importance of constant monitoring, and their probable interaction with the population (26)

The positivity of non-hematophagous bats capturedin Palmas during the time series converged with Ministry of Health data (27, 28). These animals wereimportant actors in the study of the epidemiologyof urban rabies (29), and prophylaxis measures andcorrect bat management are important actions for theprevention of this disease.

This study highlighted the importance of surveillance actions for cases of accidents involving animals potentially transmitting the rabies virus and the monitoring of prophylaxis protocols prescribed during initial care, given the number of inconsistencies identified during the first consultation. The need for continuous training of healthcare professionals for the correct administration of rabies prophylaxis stood out.

The presence of positive rabies results in samples from bats captured by animal surveillance confirmed the circulation of the virus in the urban area, generated an alert about the potential for accidents involving these animals and demonstrated the need for integrated actions between the competent institutions.

## Data Availability

The databases used were the National Notifiable Health Conditions Information System and the Palmas city Electronic Citizen Medical Record e-SUS panel. In accordance with the confidentiality and secrecy agreement for access to and use of sensitive data, both approved by the ethics committee, the authors declared that they will maintain the confidentiality, secrecy and privacy of the data contents, and that it will not be possible to disclose them, in their entirety, to any individuals not involved in the research team.
