# Supplementary material for: Evaluation of prophylaxis protocols and spatial distribution of humans wounded by bats: a descriptive study, Palmas, 2013-2023
Source: Epidemiol Serv Saude. 2026 Mar 16;35:e20250557. doi: 10.1590/S2237-96222026v35e20250557.en (PMC12995280; doi:10.1590/S2237-96222026v35e20250557.en)
Supplement: Translation

# Translation: Avaliação dos protocolos de profilaxia e distribuição espacial dos acidentes por morcegos a humanos: estudo descritivo, Palmas, 2013-2023

**DOI:** 10.1590/S2237-96222026v35e20250557.pt

**Authors:** Thais Cristine Rodrigues Freitas, Anderson Brito Soares, Benta Natânia Silva Figueiredo

**Affiliations:** 1 Fundação Escola de Saúde Pública de Palmas, Palmas, TO, Brasil; 2 Secretaria Municipal de Saúde de Palmas, Unidade de Vigilância e Controle de Zoonoses, Palmas, TO, Brasil

**Keywords:** Atendimento Antirrábico, Lyssavirus, Saúde Única, Vigilância em Saúde, Mapeamento Geográfico

## Abstract

**Objetivo:**

Avaliar as prescrições profiláticas em situações de acidentes por morcegos a humanos e a distribuição espacial dos acidentes e de morcegos capturados pela unidade de vigilância de zoonoses de Palmas, Tocantins, de 2013 a 2023.

**Métodos:**

Estudo descritivo retrospectivo das prescrições profiláticas durante o primeiro atendimento a acidentes por morcegos a humanos contidas nos prontuários eletrônicos (e-SUS) e na ficha de notificação (por meio do Sistema de Informação de Agravos de Notificação) de atendimento antirrábico. Analisaram-se as variáveis: sexo e idade do paciente, tipo de exposição, localização e caracterização do ferimento, antecedentes de tratamento antirrábico, tratamento prescrito de acordo com o protocolo vigente, indicação do soro antirrábico e interrupção do tratamento. A localização dos pacientes atendidos e de morcegos coletados para diagnóstico laboratorial foi submetida ao georreferenciamento para produção de mapas de distribuição.

**Resultados:**

De 136 casos notificados, 68,4% (93/136) dos atendimentos iniciais seguiram adequadamente o protocolo de profilaxia vigente. O Sudeste do município apresentou a maior concentração de residentes notificados (37/123). Entre 2015 e 2023, 133 morcegos foram coletados em residências e analisados quanto à presença do vírus rábico, dos quais 2,3% (3/133) foram reagentes, sendo encontrados nas regiões Noroeste, Sudoeste e Sul.

**Conclusão:**

O estudo evidenciou a relevância da vigilância epidemiológica no monitoramento dos protocolos profiláticos e atendimentos aos acidentes envolvendo morcegos, diante das inconsistências no atendimento inicial. Ressaltou-se a importância da capacitação contínua dos profissionais de saúde. A detecção do vírus em morcegos urbanos evidenciou sua circulação e reforçou a necessidade de ações interinstitucionais integradas.

**Aspectos éticos**

Esta pesquisa respeitou os princípios éticos, obtendo os seguintes dados de aprovação:

Comitê de ética em pesquisa: Fundação Escola de Saúde Pública de Palmas

Número do parecer: 6.901.095

Data de aprovação: 21/6/2024

Certificado de apresentação de apreciação ética: 77352424.4.0000.9187

Registro do consentimento livre e esclarecido: Utilização de banco de dados.

## Introdução 

A raiva é uma doença infectocontagiosa de caráter zoonótico letal que acomete o sistema nervoso central de mamíferos e tem como agente etiológico um vírus da família Rhabdoviridae e do gênero *Lyssavirus* (1). Trata-se de uma doença de extrema preocupação para a saúde pública, devido ao seu prognóstico desfavorável e à ampla diversidade genética de seu agente etiológico (2). 

A porta de entrada do vírus se dá a partir da inoculação via saliva de um mamífero infectado, principalmente através da mordedura, arranhadura ou lambedura de mucosas (3). A raiva possui quatro ciclos envolvidos na sua cadeia de transmissão: urbano, rural, silvestre terrestre e silvestre aéreo, sendo este último referente a todas as espécies de morcegos, hematófagas ou não (4).

Na América Latina, após esforços desempenhados pela Organização Pan-Americana da Saúde (OPAS) desde 1983, a incidência da doença transmitida por cães foi reduzida em aproximadamente 98%, com os morcegos sendo, em 2024, os principais reservatórios envolvidos na transmissão (5). No Brasil, seguindo o mesmo padrão, casos de raiva humana transmitidos por animais do ciclo silvestre entram em destaque, visto que o último caso humano ocasionado pela variante antigênica do vírus da raiva mantido por cães (AgV-1) foi registrado em 2015 (6,7). 

Fatores naturais, como os habitats dos animais, os reservatórios e a circulação viral, aliados aos impactos diretos das atividades antrópicas no meio ambiente, desempenham papel crucial no estudo epidemiológico da raiva (8). O Tocantins possui grande biodiversidade, marcada pela vegetação da floresta amazônica e do Cerrado. Ações de fragmentação e perda de habitats decorrentes da agricultura, como expansão urbana, mineração e construções de rodovias, podem influenciar a transmissão dessa doença no estado, uma vez que o Tocantins possui 48% das espécies de morcegos identificadas no país (9,10). 

Entre janeiro de 2010 e janeiro de 2025, 51 casos de raiva humana foram notificados no Brasil, sendo 22 (43,1%) destes causados por morcegos. Nesse período, dois óbitos foram registrados no Tocantins, nos municípios de Ponte Alta do Tocantins e Alvorada, em 2017 e 2024, respectivamente, ambos ocasionados pela variante antigênica 3 (AgV-3), mantida por morcegos hematófagos (11).

Devido à letalidade de aproximadamente 100% dos casos em humanos e à ausência de tratamento específico, evidencia-se a importância do correto desempenho e execução dos protocolos de profilaxia pré, pós e reexposição ao vírus rábico (12). A profilaxia baseia-se no potencial risco de infecção pelo vírus e deve ser adotada mediante a análise dos casos, a anamnese completa dos pacientes e o registro dos dados do agravo (13). 

O protocolo profilático atualmente preconizado pelo Ministério da Saúde consiste na aplicação de vacina antirrábica com ou sem o uso do soro antirrábico ou da imunoglobulina humana antirrábica, a depender da situação observada. Todos os casos de acidentes por morcegos e demais animais silvestres devem ser considerados acidentes graves, com o acompanhamento e a monitoração desses pacientes, para que casos de abandono de tratamento e de administração profilática insuficiente sejam evitados (14).

Esta pesquisa teve como objetivo avaliar as prescrições profiláticas em situações de acidentes por morcegos a humanos e a distribuição espacial dos acidentes e de morcegos capturados pela unidade de vigilância de zoonoses de Palmas, Tocantins, de 2013 a 2023.

## Métodos 

### Delineamento do estudo

Tratou-se de estudo descritivo retrospectivo sobre as medidas profiláticas prescritas para pacientes notificados por acidentes por morcegos. Utilizou-se o georreferenciamento das respectivas residências e da localização de captura de morcegos em Palmas, Tocantins, de janeiro de 2013 a dezembro de 2023.

### Contexto 

O município faz parte da Amazônia Legal e fica localizado a 260 metros acima do nível do mar, no centro-sul de Tocantins. Possui como limite a oeste o lago formado pela usina hidrelétrica de Luís Eduardo Magalhães e a leste as serras do Carmo e do Lajeado. Em 2022, com 302.692 habitantes, possuía área territorial de 2.227,329 km^2^ e densidade demográfica de 135,90 habitantes/km^2^. As temperaturas são elevadas ao longo do ano com baixa amplitude térmica e distribuição sazonal bem definida, com período chuvoso de outubro a abril, marcado por ventos fracos a moderados, e período seco de maio a setembro. 

A cidade é caracterizada por áreas de vegetação no perímetro urbano, que formam um corredor ecológico, com presença de parques e áreas de reserva. Administrativamente, o município possui cinco regiões: Sul, Sudoeste, Sudeste, Noroeste e Nordeste (15,16) (Figura 1). 

**Figura 1:** Regiões administrativas do perímetro urbano. Palmas, 2024

### Fonte de dados

Foram coletados dados a partir da exportação das fichas de atendimento antirrábico humano notificadas em nível municipal, especificamente para acidentes por morcegos, no Sistema Nacional de Agravos e Notificações. Para o acréscimo de informações pertinentes ao atendimento dos pacientes, foram utilizadas informações contidas no Prontuário Eletrônico do Cidadão do painel e-SUS e da Secretaria Municipal de Saúde. Atendimentos registrados entre 2013 e 2015 foram analisados conforme informações e observações das fichas de notificação e, a partir de 2016, foram utilizados dados do prontuário eletrônico.

Os dados referentes à localização de capturas de morcegos registrados pela vigilância animal da Secretaria Municipal de Saúde e resultados dos exames para detecção do vírus rábico do período 2015-2023 foram obtidos junto à unidade de vigilância e controle de zoonoses de Palmas.

### Variáreis e análise de dados 

Os dados da condução dos atendimentos profiláticos foram exportados com auxílio do software TabWin (versão 4.13) e, posteriormente, organizados em planilhas e submetidos à análise descritiva. Prontuários com preenchimento incorreto e ausência de endereço completo, que impossibilitava o georreferenciamento, foram excluídos. 

Para análise das condutas profiláticas prescritas, foram analisadas as variáveis: sexo e idade do paciente, tipo de exposição, localização e caracterização do ferimento, antecedentes de tratamento antirrábico, tratamento prescrito de acordo com o protocolo vigente, indicação do soro antirrábico e interrupção do tratamento (13).

Para georreferenciamento, foram utilizadas informações quanto ao bairro, ao logradouro, ao número, ao complemento e zona dos pacientes notificados e à latitude e longitude dos morcegos capturados. Para endereços incompletos, utilizou-se a metodologia de georreferenciamento do centro do logradouro. 

As informações das localidades foram organizadas em planilhas e exportadas para o software QGis (versão 3.28.11-Firenze). Este possibilitou a elaboração de mapas de calor por meio do estimador de densidade Kernel, ferramenta que calculou a densidade de pontos no espaço geográfico, onde, por meio de gradações de cor, áreas com maior concentração de eventos específicos ficaram em evidência (17).

Os resultados de positividade de amostras para o vírus rábico foram georreferenciados e submetidos à análise descritiva.

## Resultados 

No período analisado, foram registradas 136 notificações de atendimento antirrábico humano relacionadas a acidentes por morcegos em Palmas (média anual=12,36 casos). Observou-se que o ano de 2021 concentrou o maior número de notificações, correspondendo a 16,2% (22/136) do total (Figura 2). Foram provenientes de zona urbana 94,9% (129/136) das notificações. Pacientes do sexo masculino foram os mais acometidos e representaram 58,8% (80/136) dos casos, com destaque de 35,3% (48/136) para a faixa etária 20-34 anos, seguida das faixas 35-49 anos com 29,4% (40/136), 50-64 anos com 18,4% (25/136), 15-19 anos com 5,1% (7/136), 65-79 anos com 3,7% (5/136), 1-4 anos com 2,9% (4/136), 5-9 anos com 2,2% (3/136), 10-14 anos com 2,2% (3/136) e 80+ anos com 0,7% (1/136) das notificações.

**Figura 2:** Números absolutos de notificações de atendimento antirrábico humano por acidentes por morcegos de 2013 a 2023 (n=136) e de morcegos coletados de 2015 a 2023 (n=133). Palmas, 2024

O tipo de acidente com risco de exposição ao vírus rábico mais comum foi por mordedura (81,6%), ocorrendo principalmente na região de mãos e pés (66,9%). Os ferimentos foram descritos, em sua maioria, como únicos em 74,3% e profundos em 53,0% dos casos (Tabela 1).

**Tabela 1:** Características dos acidentes relacionados às notificações de atendimento antirrábico humano por acidentes por morcegos de 2013 a 2023. Palmas, 2024 (n=136)

Características	n (%)
**Tipo de exposição ao vírus rábico**	
Contato indireto	2 (1,5)
Arranhadura	17 (12,5)
Lambedura	3 (2,2)
Mordedura	111 (81,6)
Outro	4 (2,9)
Localização	
Mucosa	2 (1,5)
Cabeça/pescoço	12 (8,8)
Mãos/pés	91 (66,9)
Tronco	9 (6,6)
Membros superiores	9 (6,6)
Membros inferiores	12 (8,8)
Ferimento	
Único	101 (74,3)
Múltiplo	25 (18,4)
Sem ferimento	10 (7,4)
Tipo de ferimento	
Profundo	72 (53,0)
Superficial	53 (39,0)

A análise dos atendimentos iniciais registrados mostrou que 68,4% (93/136) dos casos seguiram adequadamente o protocolo de profilaxia vigente. Os demais (43/136) apresentaram prescrições insatisfatórias no primeiro atendimento: ausência da prescrição de soro antirrábico em 44,2% (19/43), adoção de protocolo obsoleto em 27,9% (12/43), prescrição de doses insuficientes da vacina antirrábica em 9,3% (4/43), protocolo de reexposição inadequado em 7,0% (3/43), ausência de informações de prescrição em 7,0% (3/43), observação do animal em 2,3% (1/43) e dispensa de tratamento em 2,3% (1/43).

Cem por cento das prescrições classificadas como insatisfatórias sofreram intervenção da vigilância epidemiológica municipal, por meio da Área Técnica de Atendimento Antirrábico Humano da Secretaria Municipal de Saúde de Palmas, para adequação ao protocolo vigente.

Do total de pacientes notificados, 10,3% (14/136) abandonaram o tratamento prescrito. Por sua vez, 1,5% (2/136) dos casos foram encerrados como abandono de tratamento na ficha de notificação, embora tenham concluído o esquema. A busca ativa dos pacientes classificados como abandono de tratamento foi realizada em 100,0% dos casos.

Foram localizados e interpretados, conforme o estimador de densidade Kernel, 123 endereços (Figura 3). A região administrativa Sudeste apresentou maior concentração de residentes notificados (37/123), seguida das regiões Sul (29/123), Noroeste (24/123), Sudoeste (19/123) e Nordeste (14/123). 

**Figura 3:** Notificações de pacientes agredidos por morcegos de 2013 a 2023, com maior quantidade nas áreas em vermelho. Palmas, 2024 (n=123)

Entre 2015 e 2023, 133 morcegos foram coletados em residências do município e analisados quanto à presença do vírus rábico, nos quais 2,3% (3/133) apresentaram resultados positivos, sendo 1 no Noroeste em 2018, 1 no Sudoeste e 1 no Sul em 2019. 

Trinta e cinco morcegos coletados foram provenientes da região administrativa Sudeste; 31 do Sudoeste; 24 do Noroeste; 21 do Nordeste; 14 do Sul; e 8 da zona rural. Com relação aos abrigos de morcegos registrados no perímetro urbano da capital, 2 foram georreferenciados, ambos pertencentes à região Sudoeste. Observou-se a sobreposição entre morcegos coletados em áreas residenciais e nas residências onde foram notificados pacientes agredidos por esses animais, de modo que a maior concentração foi encontrada no Sudeste em ambos os casos (Figura 4).

**Figura 4:** Distribuição dos pontos de coleta de morcegos para diagnóstico laboratorial e dos abrigos cadastrados, sobrepostos ao mapa de calor dos endereços de pacientes agredidos por morcegos no período 2013-2023. Palmas, 2024 (n=133)

## Discussão 

A análise dos prontuários de atendimento antirrábico humano devido a acidentes por morcegos evidenciou predominância de pacientes do sexo masculino, faixa etária a partir dos 20 anos e residentes da zona urbana. Esse padrão sociodemográfico corroborou a literatura, que descreveu perfil semelhante nos atendimentos realizados em capitais brasileiras (18).

Ao avaliar as prescrições do protocolo profilático realizado durante o primeiro atendimento, notou-se que os protocolos foram adequados em 68% dos casos. Todavia, uma parcela apresentou divergências, uma vez que tais casos deveriam ter sido conduzidos como acidentes graves, sendo a sorovacinação primordial em situações de pós-exposição (13). 

Embora a maioria das prescrições seguisse adequadamente as recomendações em situações de acidentes com risco de transmissão do vírus da raiva, foram relatados equívocos no atendimento inicial quando comparados ao protocolo vigente, como o uso exclusivo de vacina ou ainda a dispensa de tratamento (19,20). Foram descritos casos de interrupções e ou abandonos do esquema profilático, bem como registros duplicados em diferentes unidades de saúde, o que pôde indicar fragilidades durante o preenchimento das notificações e do cumprimento do protocolo vigente (20). Tais dados foram importantes para o planejamento e execução do serviço ofertado, uma vez que, entre 2002 e 2012, em 80% dos óbitos de raiva humana ocasionados por mamíferos silvestres no país não houve nenhum tipo de profilaxia antirrábica ou esta foi administrada inadequadamente (21).

Evidências apontaram que a elevada rotatividade de médicos e enfermeiros, associada à insuficiência de ações educativas permanentes, foi fator contribuinte para a insegurança na tomada de decisão terapêutica e na prescrição por parte dos profissionais de saúde (22,23).

Neste estudo, verificou-se que a vigilância epidemiológica atuou de forma efetiva, adequando oportunamente todos os protocolos e realizando a busca ativa de todos os casos de abandono ou interrupção do tratamento. Tais achados reforçaram a relevância da vigilância, ao mesmo tempo em que evidenciaram a necessidade de fortalecer a capacitação contínua dos profissionais de saúde e a integração entre a vigilância animal e humana. Isso permitiu incorporar estratégias intersetoriais, alinhadas à perspectiva “Uma Só Saúde”, a fim de garantir maior coerência e eficiência nas ações de resposta.

Devido às mudanças no perfil epidemiológico da raiva humana, surgiu a necessidade de estratégias de vigilância aprimoradas para prevenção desse agravo, principalmente em áreas com maior risco de exposição a morcegos (6). Das 182 espécies de morcegos registradas no Brasil, 46% foram encontradas em ambientes urbanos. Recursos favoráveis nesse ambiente para a manutenção de colônias, como a facilidade de encontro de abrigos artificiais e a disponibilidade de alimento, demonstraram a predisposição para a formação de colônias nessas localidades (24). Por sua vez, a predominância de pacientes agredidos residentes da zona urbana de Palmas reforçou também o processo de expansão territorial da capital, favorecendo a sobreposição entre ambientes antrópicos e áreas de abrigo e alimentação de morcegos (25). 

As espécies de morcegos insetívoros são atraídas pela iluminação das cidades, por concentrarem insetos em seus arredores, além das espécies frugívoras, fitófagas e nectarívoras, as quais são atraídas pela arborização (24). Relatou-se a presença de uma grande colônia de morcegos insetívoros da família Molossidae na área urbana de Palmas, em um abrigo localizado na ponte Governador José Wilson Siqueira Campos – o mesmo abrigo foi georreferenciado neste trabalho (Figura 4). Tal informação despertou a necessidade de se considerar a existência desses animais no meio urbano, a sua sustentabilidade popu­lacional, a importância do monitoramento constante e a provável interação com a po­pulação (26)

A positividade dos morcegos não hematófagos coletados em Palmas durante a série histórica convergiu com os dados apontados pelo Ministério da Saúde (27,28). Esses animais foram atores importantes no estudo da epidemiologia da raiva urbana (29), sendo as medidas de profilaxia e manejo correto de morcegos ações importantes para a prevenção dessa doença.

Este estudo evidenciou a importância das ações de vigilância dos casos de acidentes por animais potencialmente transmissores do vírus da raiva e do monitoramento dos protocolos profiláticos prescritos no atendimento inicial, visto a quantidade de inconsistências identificadas durante o primeiro atendimento. Destacou-se a necessidade da capacitação contínua de profissionais de saúde para a correta administração da profilaxia antirrábica.

A presença de resultados positivos nas amostras de morcegos capturados pela vigilância animal comprovou a circulação do vírus na área urbana, gerou um alerta para o potencial de acidentes envolvendo esses animais e demonstrou a necessidade de ações integradas entre as instituições competentes.

## Data Availability

Os bancos de dados utilizados foram o Sistema Nacional de Agravos e Notificações e o Prontuário Eletrônico do Cidadão no painel e-SUS de Palmas. De acordo com o registro de confidencialidade e sigilo, para acesso e utilização de dados sensíveis, ambos aprovados pelo comitê de ética, os autores declararam manter a confidencialidade, o sigilo dos dados e a privacidade de seus conteúdos, não sendo possível repassá-los, em sua íntegra, a quaisquer indivíduos não envolvidos na equipe da pesquisa.
